# Kikuchi-Fujimoto Disease in a 30-Year-Old Caucasian Female

**DOI:** 10.1155/2009/901537

**Published:** 2009-12-20

**Authors:** David J. Archibald, Matthew L. Carlson, Ray O. Gustafson

**Affiliations:** Department of Otorhinolaryngology, Mayo Clinic, 200 First Street SW, Rochester, MN 55905, USA

## Abstract

Kikuchi-Fujimoto disease is a rare, self-limited, histiocytic, necrotizing lymphadenitis first described in Japan in 1972. Necrosis of lymph node tissue is caused by apoptosis and may be virally induced. It commonly presents with cervical lymphadenitis and fever. Despite its low incidence, Kikuchi-Fujimoto disease should be considered in patients with persistent lymphadenopathy. Originally thought to occur only in young Asian women, it is now recognized in other geographic regions. We report a 30-year-old white woman with Kikuchi-Fujimoto disease. We discuss the clinical features, differential diagnosis, radiography, pathology, and outcome.

## 1. Introduction

Kikuchi-Fujimoto disease (KFD), also known as histiocytic necrotizing lymphadenitis, is an uncommon, self-limited, benign, systemic lymphadenitis of unknown etiology. By the late 1960s, members of the Pathology Reference Center [1] recognized an unusual form of necrotizing lymphadenitis that, in several instances, had been misdiagnosed as malignant lymphoma. In 1972, Japanese pathologists Kikuchi [2] and Fujimoto et al. [3] independently described the disease as a “lymphadenitis showing focal reticulum cell hyperplasia with nuclear debris and phagocytosis” and “cervical subacute necrotizing lymphadenitis,” respectively. Although KFD was initially characterized as occurring exclusively in the cervical lymph nodes of young Asian women, it has been observed in patients of any age, sex, and race, and it can involve nodal and extranodal locations.

## 2. Report of a Case

A 30-year-old white woman presented to the emergency department with a 4-day history of a left anterior cervical mass. She reported that swelling in her upper neck had been waxing and waning. In the 36 hours before her presentation, the swelling had decreased the range of motion in her neck. The mass was causing severe pain and tenderness over the sternocleidomastoid muscle, and she also had mild trismus. She reported intermittent fevers up to 39°C and denied any airway compromise or otalgia. She was a nonsmoker with an unremarkable medical history, except for Hashimoto thyroiditis. Her medical regimen included levothyroxine sodium and azithromycin. 

**Figure 1:**
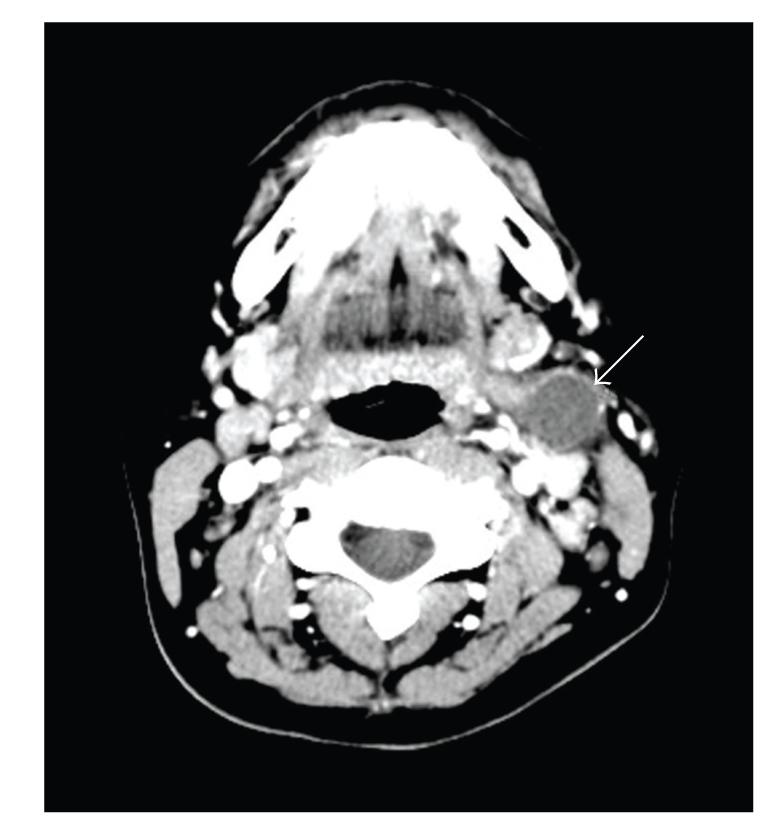
Axial contrast-enhanced CT. A 2 cm well-circumscribed, low attenuation mass (arrow) is present at the expected location (jugulodigastric (Level 2) lymph node or second branchial cleft cyst).

On physical examination, the patient was febrile, and there was obvious left cervical lymphadenopathy with warmth and tenderness over the neck. Nasal and oral examination results were unremarkable. Her white blood cell count was 7.2 × 10^9^/L (reference range, 3.5–10.5 × 10^9^/L) and consisted of 77% neutrophils, 8% monocytes, 14% lymphocytes, less than 1% basophils, and less than 1% eosinophils. Her hemoglobin level was 8.4 g/dL, (reference range, 12.0–15.5 g/dL), and her platelet count was 157 × 10^9^/L (reference range, 150–450 × 10^9^/L). A chest radiograph was negative for any masses or lymphadenopathy. A computed tomographic (CT) scan revealed a 2 cm mass in the left jugular digastric region (level 2) with peripheral enhancement (Figure 1). Scattered lymph nodes along the left jugular chain were also enlarged.

**Figure 2:**
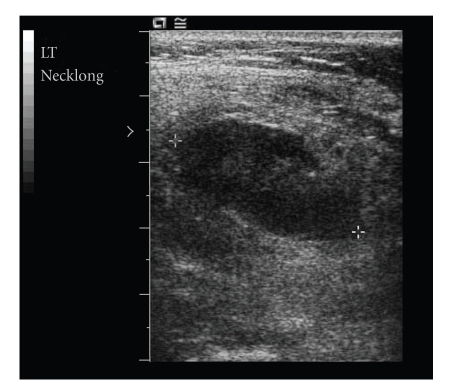
Ultrasonogram of the largest lymph node at the site of cervical swelling. The lymph node was a solid mass measuring 2.6 × 3.1 cm.

The infectious disease consulting service recommended an ultrasonographically guided needle aspiration of the presumptive cystic fluid. When ultrasonography indicated the mass was solid (Figure 2), the patient instead underwent an excisional biopsy. During surgery, a friable mass was identified and removed. The biopsy tissue was evaluated through frozen-section pathology, special lymphoma stains, and flow cytometry and showed a necrotizing lymphadenitis with foci of mottled tissue and mononuclear cells in the paracortical region of the lymph node. The patient continued to have spiking fevers for several days; however, her fevers were generally less intense (her temperature did not exceed 38.5°C).

Ten days after the biopsy, the patient reported ear and neck pain but denied any recurrent fevers. She was advised to take ibuprofen for 2 to 3 days and to return for reimaging if the pain persisted. The patient′s pain eventually resolved, and she had no further complications.

## 3. Discussion

### 3.1. Demographics

The incidence of KFD is unknown. It has been observed in patients aged 19 months to 75 years (typically 25–29 years old) [4]. KFD is 3 to 4 times more common in women than in men [4]. Although patients can be of any race, most case reports are from Asia, where KFD was first described. This geographic predominance may arise from several HLA class II alleles (*HLA-DPA1* and *HLA-DPB1*) that are more prevalent in Asian KFD patients [5]. Furthermore, the *HLA-DPB1* allele appears with considerably higher frequency in Asians when compared with whites [5]. 

### 3.2. Etiology

The etiology of KFD has not been identified. Necrosis of the lymph nodes appears to be caused by apoptosis [6]. Recent studies suggest the primary mechanism of KFD necrosis involves perforin, a cytolytic protein. The Epstein-Barr virus and the human herpesvirus have also been identified as possible causes of apoptosis [7].

### 3.3. Signs and Symptoms

Despite the low incidence, KFD should be considered in patients with persistent lymphadenopathy, and an early diagnosis from biopsy findings can prevent unnecessary investigations and treatments. Most patients (80%) present with cervical lymphadenitis, although any lymph node region may be involved [8]. Neck involvement is usually unilateral. Cervical lymphadenopathy is common in the posterior cervical triangle and jugular carotid chain [9]. Other affected areas include the axillary (14%) and supraclavicular (12%) nodal chains [8]. The lymphadenopathy may be firm and sometimes painful. It is usually isolated, but 1% to 20% of patients have generalized lymphadenopathy [8]. Fever is a primary symptom in 30% to 50% of patients; there are few additional symptoms [10]. Less common symptoms include hepatosplenomegaly, headache, weight loss, malaise, chills, night sweats, and gastrointestinal complaints. In a small subset of patients, cutaneous symptoms have been noted. Skin lesions have been described as maculopapular, morbilliform or rubella-like, and urticarial. Disseminated erythema has also been reported [10].

### 3.4. Imaging

Radiographic findings specific to KFD have not been established, and reports of KFD in the medical literature have largely focused on the disease pathology. Chest radiography should be included in the diagnostic evaluation to eliminate the possibility of malignancy or tuberculosis. CT and ultrasonography are common and often helpful before obtaining a biopsy specimen. Ultrasonography frequently shows lymph nodes with a hypoechoic center and a hyperechoic rim [11, 12]. CT findings generally mimic those of lymphoma; however, lymph nodes in patients with KFD are not as large as those in patients with lymphoma [13, 14]. 

The radiographic diagnosis of KFD is broad and includes Hodgkin and non-Hodgkin disease, metastatic tumor, tuberculosis, nontuberculous mycobacterial infection, SLE, human immunodeficiency virus infection, infectious mononucleosis, cat-scratch disease, mucocutaneous lymph node syndrome (Kawasaki disease), and toxoplasmosis. In 1 series, 29% of patients with KFD were initially misdiagnosed as having Hodgkin or non-Hodgkin disease [15].

### 3.5. Laboratory Studies

There are no specific assays available to confirm the diagnosis of KFD, but laboratory tests are used to eliminate other causes of cervical lymphadenopathy. Peripheral blood tests indicate 25.0% to 58.3% of KFD patients had leukopenia, and 25.0% to 31.1% of patients had atypical lymphocytes [16]. Extensive testing for infectious agents is usually not diagnostic, although there are reports of KFD associated with the Epstein-Barr virus [4]. Few patients diagnosed with KFD had positive laboratory test results for SLE and later developed that disease. Nevertheless, patients with KFD are recommended to undergo long-term monitoring for the development of SLE [15], even if the frequency of follow-up visits and duration of the follow-up period are not specifically defined.

### 3.6. Diagnosis

Patients suspected of having KFD often undergo fine-needle aspiration before an excisional biopsy is performed. Aspiration was not performed on our patient because ultrasonography indicated that the mass was solid. 

**Figure 3:**
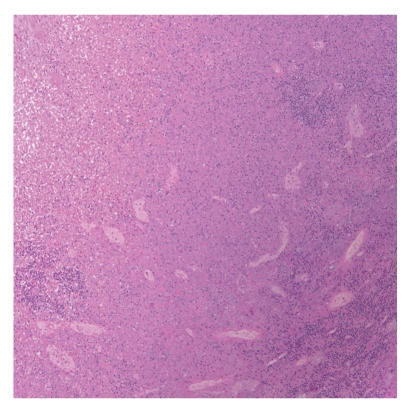
Distribution of necrotic foci (mottled tissue) and mononuclear cells in the paracortical region of a lymph node (Hematoxylin-eosin stain, original magnification ×10.).

**Figure 4:**
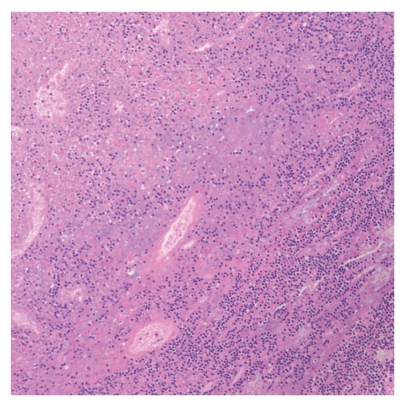
Necrotizing lymphadenitis of a lymph node with associated perinodal necrosis. Some mononuclear cells may represent macrophages phagocytosing nuclear debris (Hematoxylin-eosin stain, original magnification ×20.).

The definitive diagnosis of KFD is made through lymph node excision biopsy and histologic examination. There are several classic histologic features of KFD. The affected lymph nodes have patchy necrotizing regions (the degree of necrosis varies widely among patients), mainly in the paracortical areas (Figure 3). They often contain well-circumscribed areas of eosinophilic fibrinoid material with a substantial degree of karyorrhexis. Transformed lymphocytes (immunoblasts) may surround the necrotic areas, creating a characteristic mottled appearance at low magnification. Nuclear debris (“nuclear dust”) is evenly scattered throughout the necrotic areas and is associated with atypical mononuclear cells, which may be macrophages phagocytosing the debris (Figure 4). Another consistent histologic observation is the absence of granulocytes and few or no plasma cells [15]. Although histologic lymph node features are distinct in KFD, some overlaps exist, especially with SLE. Effective communication between the pathologist and otolaryngologic surgeon is critical for correct disease identification.

The differential diagnosis of a slow-growing neck mass is extensive and includes malignant lymphoma, systemic lupus erythematosus (SLE), Hodgkin disease, toxoplasmosis, metastatic carcinoma, infectious mononucleosis, acquired immunodeficiency syndrome, cat-scratch disease, and angioimmunoblastic lymphadenopathy [15]. To differentiate KFD from lymphoma, KFD features an increased number of slightly enlarged lymph nodes, whereas lymphoma usually produces moderately to markedly enlarged lymph nodes [17]. Excised lymph nodes of KFD patients are typically smaller than 3 cm. 

### 3.7. Outcomes

In 80% of patients, KFD is self-limited and resolves within 1 to 6 months without specific treatment [18]. Patients with extensive systemic manifestations, pyrexia, lymphadenopathic pain, or a combination of these symptoms have been treated with steroidal or nonsteroidal anti-inflammatory medications, and patients with advanced KFD may benefit from treatment with systemic prednisone [10]. There are 3 KFD-associated deaths in literature, and patients with recurring KFD have been reported in some studies [18]. In 1 investigation, the recurrence rate of KFD was 3.3% over 2 years [16].

## 4. Summary

KFD is a self-limited and typically benign lymphadenitis of unknown etiology now recognized in multiple geographic regions and races. The clinical suspicions of the otolaryngologic surgeon, in conjunction with the pathology findings, are critical for an accurate diagnosis. Understanding the characteristic clinical presentation and histologic findings of KFD help exclude malignant disorders and nonneoplastic conditions that require specific therapy.

## Abbreviations

CT: Computed tomographic, computed tomography
KFD: Kikuchi-Fujimoto disease
SLE: Systemic lupus erythematosus
